# Extended Use of the Omnipod 5 Automated Insulin Delivery System in Adults With Type 1 Diabetes: 12‐Month Extension of a Randomized Controlled Trial

**DOI:** 10.1002/edm2.70321

**Published:** 2026-08-31

**Authors:** Eric Renard, Ruth S. Weinstock, Alfred Penfornis, Jean‐Pierre Riveline, Charles Thivolet, Trang T. Ly, for the OP5‐003 Research Group, Eric Renard, Anne Farret, Orianne Villard, Manal Al Masri, Ruth S. Weinstock, Suzan Bzdick, Alfred Penfornis, Catherine Petit, Marcelle Siadoua, Jean‐Pierre Riveline, Jean‐François Gautier, Tiphaine Vidal‐Trecan, Jean‐Baptiste Julla, Charline Potier, Djamila Bellili, Charles Thivolet, Sylvie Villar Fimbel, Redhouane Hami, Kaisa Kivilaid, Trang T. Ly, Bonnie Dumais, Todd Vienneau, Lauren M. Huyett, Lindsey R. Conroy

**Affiliations:** ^1^ Department of Endocrinology and Diabetes, Montpellier University Hospital; INSERM CIC1411 Clinical Investigation Center, Institute of Functional Genomics, CNRS, INSERM University of Montpellier Montpellier France; ^2^ Department of Medicine SUNY Upstate Medical University Syracuse New York USA; ^3^ Hospital Center Sud Francilien, Paris‐Saclay University Corbeil‐Essonnes France; ^4^ Lariboisière Hospital, Paris Cité University Paris France; ^5^ DIAB‐eCARE Diabetes Center Lyon France; ^6^ Insulet Corporation Acton Massachusetts USA

**Keywords:** automated insulin delivery, extension study, France, Omnipod 5, randomized controlled trial, Type 1 diabetes

## Abstract

**Background:**

The Omnipod 5 Automated Insulin Delivery (AID) System is safe and effective for individuals managing Type 1 diabetes (T1D). Longer‐term studies may provide additional evidence of sustained effectiveness and safety of AID system use in T1D.

**Methods:**

This 12‐month extension study was conducted following a multicenter randomized controlled trial (RCT) where participants used either AID (Omnipod 5) or standard therapy (current non‐automated pump therapy) for 13 weeks. Participants in France (*n* = 76) could transition to or continue with AID for an additional 12 months. Glycemic, safety, and psychosocial outcomes during or at the end of the extension phase were compared with baseline or end of RCT, as appropriate.

**Results:**

Seventy‐five participants enrolled in the extension phase. From RCT baseline to the end of the extension phase, time in range 70–180 mg/dL increased by 17.9% (*p* < 0.0001) or 4.3 h/day to 62.3%. Time above range > 180 mg/dL and mean sensor glucose decreased by 17.7% and 27.8 mg/dL (both *p* < 0.0001), respectively. HbA1c decreased from 8.33% to 7.18% (−1.14%; *p* < 0.0001). Glycemic improvements were maintained for those continuing with AID from the RCT intervention group and for those transitioning to AID from standard therapy. Diabetes Quality of Life‐brief and Hypoglycemia Confidence Scale scores were maintained or improved at 6 and 12 months compared to RCT baseline. Adverse events were infrequent (12 per 100 person‐years).

**Conclusions:**

Findings support the RCT results, demonstrating safety and sustained improvements in glycemic and psychosocial outcomes with the Omnipod 5 System in adults in France with T1D over 12 months.

**Trial Registration:** ClinicalTrials.gov NCT05409131

## Graphical abstract

In this 12‐month extension of a randomized controlled trial, adults in France with Type 1 diabetes using the Omnipod 5 Automated Insulin Delivery System sustained a 17.9% increase in time in range (4.3 h/day) and a 1.14% HbA1c reduction, with preserved quality of life and few adverse events.

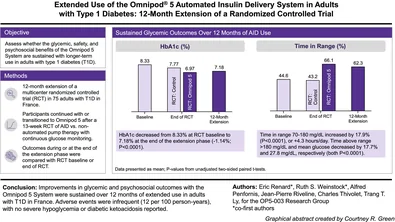

## 1 Introduction

Most people with Type 1 diabetes are not meeting recommended clinical targets (HbA1c < 7% [53 mmol/mol]), placing them at an increased risk for serious adverse health outcomes as well as higher social and economic burdens [1]. Automated insulin delivery (AID) systems, a relatively recent technological advancement, use closed‐loop control algorithms combined with an insulin pump and continuous glucose monitoring (CGM) to optimize glucose management by automatically adjusting insulin delivery [2]. AID systems have been shown to improve glycemic outcomes and reduce the burden of diabetes management [3]. Given their demonstrated efficacy, consensus guidelines recommend AID systems for all individuals with insulin‐deficient diabetes [2, 4].

The Omnipod 5 Automated Insulin Delivery System (Omnipod 5 System; Insulet Corporation, Acton, MA), the first tubeless AID system, uses a novel algorithm and customizable glucose targets. The Omnipod 5 System has been shown to be safe and effective for the treatment of Type 1 diabetes in single‐arm clinical trials for individuals aged 2 to 70 years [5, 6, 7, 8], in subsequent extension trials [7, 8], in real‐world analyses of youth and those ≥ 2 years old [9, 10], and more recently in a 13‐week multicenter, parallel‐group randomized controlled trial (RCT) of adults 18 to 70 years [11]. The RCT, conducted in centers in the US and France, found that the Omnipod 5 System outperformed non‐automated pump therapies with CGM, showing a greater percentage of time in range (70–180 mg/dL) (61.2% vs. 43.8%, *p* < 0.0001), equivalent to 4.2 more hours per day, along with less time spent in hypoglycemic (< 70 mg/dL) or hyperglycemic (> 180 mg/dL) ranges [11]. These findings, primarily from participants with baseline HbA_1c_ levels ≥ 8% (64 mmol/mol), were concomitant with a greater proportion of participants with improved patient‐reported outcomes among those using the Omnipod 5 System and very low rates of adverse events [11]. Longer‐term follow‐up can provide further evidence of the durability of the observed effectiveness and safety of the Omnipod 5 System. The objective of this study was to gain additional insights into the use of the Omnipod 5 System in adults with Type 1 diabetes in France.

## 2 Methods

### 2.1 Study Design

This study was a 12‐month extension to a 13‐week multicenter (US and France), parallel‐group RCT (ClinicalTrials.gov: NCT05409131) [11]. Briefly, the RCT included a 2‐week standard therapy baseline period, followed by the 13‐week trial in which participants (*n* = 194) were randomized to either Omnipod 5 AID System (CE‐marked) or control (current pump therapy with CGM). Additional details on the RCT were previously published [11]. Participants from the 4 sites in France were then presented with the opportunity to continue using AID or transition to AID for this 12‐month extension phase. The protocol was approved by central and local institutional review boards and ethics committees. Written informed consent to participate in the extension phase was obtained from all participants. A medical monitor provided trial oversight.

Briefly, the Omnipod 5 System, which is described in detail elsewhere [12], consists of an insulin‐filled tubeless on‐body pump (Pod) with a built‐in AID algorithm and the Omnipod 5 application (on a provided locked‐down Android phone or a compatible personal smartphone), which controls the Pod via Bluetooth wireless technology. An interoperable CGM is required to use AID, and the Dexcom G6 CGM was used for this study. The Omnipod 5 System, which was investigational in France during the RCT phase, received the CE‐mark on September 19, 2022. The Omnipod 5 System is currently FDA cleared and CE marked for people ≥ 2 years of age with Type 1 diabetes and is FDA cleared for adults ≥ 18 years of age with Type 2 diabetes. All participants used the System in Automated Mode, which delivers insulin micro‐boluses every 5 min to bring glucose levels towards a selected target (customizable in 10 mg/dL increments between 110 and 150 mg/dL) based on CGM‐measured glucose values. Participants were trained on the use of the Omnipod 5 System.

### 2.2 Participants

The extension study was conducted between November 27, 2023 and January 16, 2025. To be eligible for the original RCT, participants had to be between 18 and 70 years, have had Type 1 diabetes for at least 1 year, and have an HbA_1c_ of 7.0%–11.0% (53–97 mmol/mol). Only participants who had been using non‐automated pump therapy for at least 3 months prior to RCT screening were eligible, excluding individuals using AID devices or predictive low‐glucose suspend. Complete eligibility criteria for the RCT are provided in Table S1. In addition, participants who withdrew from the RCT for any reason were not eligible for the extension phase.

During the 12 months of the extension phase, there were eight follow‐up visits conducted either in person or over the phone (Table S2). Five of these follow‐up visits were conducted for all participants, during which vital signs, HbA_1c_, and weight were assessed, adverse events and device complaints were reviewed, pump, CGM, and blood glucose/ketone data were evaluated, and patient‐reported outcome measures were completed at select visits; the final visit included study closure procedures and return of study devices. The other three follow‐up visits for Omnipod 5‐naïve participants from the RCT control group were conducted either in person or by phone to provide additional monitoring and support.

### 2.3 Outcomes

All outcomes were considered exploratory with no pre‐specified hypotheses. To characterize System utilization during the extension phase, cumulative use was assessed using device upload data. The proportion of time spent using Omnipod 5 in either Manual Mode or Automated Mode, the proportion of time spent in Automated Mode, and the distribution of selected glucose target settings were summarized descriptively over the study period. Performance endpoints were compared to baseline or end of RCT measures. Baseline consisted of a 2‐week standard therapy period prior to the RCT. These endpoints included: HbA_1c_ at 3, 6, 9, and 12 months, change in HbA_1c_, proportion of participants with HbA_1c_ < 7% and < 8%, percentage of time < 54 mg/dL, < 70 mg/dL, > 180 mg/dL, and 70–180 mg/dL (time in range), percentage of participants with ≥ 70% time in range, percentage of participants with < 4% time below range < 70 mg/dL, mean glucose, change in body mass index (BMI) at 6 and 12 months, and insulin usage.

At 6 and 12 months (or upon withdrawal), participants were given the Diabetes Quality Of Life‐brief questionnaire to assess the relative burden of intensive diabetes treatment [13] and the Hypoglycemia Confidence Scale to measure hypoglycemia unawareness, frequency, severity, and impact [14]. For the Hypoglycemia Confidence Scale (HCS), a score of ≥ 3 is indicative of relatively high confidence in managing hypoglycemia [14].

Adverse events were monitored on an ongoing basis. Reportable adverse events included severe or prolonged hyperglycemia, hyperglycemia involving ketosis, diabetic ketoacidosis, severe hypoglycemia, any event affecting a participant's ability to adhere to study procedures or requiring a visit to a hospital emergency department, and adverse device events.

### 2.4 Statistical Analysis

Data from all participants were combined into a modified intention‐to‐treat database regardless of the original RCT treatment group. A minimum of 2016 CGM readings (equivalent to 7 days) over the 12 months was required for inclusion in glycemic analyses, a threshold chosen to retain the maximum number of participants while ensuring stable CGM‐derived estimates; no participants were excluded on this basis. No formal hypothesis testing was performed. Outcomes were analysed using descriptive statistics with continuous variables summarized using count, mean, median, SD, range, and categorical variables summarized using frequencies and percentages. Comparisons were done using unadjusted two‐sided paired *t*‐tests and two‐sided Wilcoxon signed rank tests for non‐parametric analyses. Incidence rates of adverse events were calculated per 100 person‐years. No imputations for missing data were made. *p*‐values with a two‐sided significance level of 5% were considered statistically significant. Analyses were performed using SAS 9.4 statistical software.

## 3 Results

Seventy‐five of the 76 participants from France enrolled in the RCT consented and participated in the extension phase (Figure S1). One person withdrew from the extension study before the final visit because they moved out of France. No scheduled visits were missed by the remaining participants; however, protocol deviations related to testing not performed resulted in missing data for some study outcomes at select time points. Data from all 75 participants (including 52 who used the Omnipod 5 System in the RCT and 23 who did not) were included in the analyses. Participants were 60% female with a mean age of 40 ± 13 years at baseline (Table 1). Most participants (74/75; 98.7%) were using a non‐automated Omnipod pump prior to the study.

**TABLE 1:** Characteristics of participants at baseline (modified intention‐to‐treat dataset).

Characteristic	All participants (*n* = 75)
Age (years)	
Mean ± SD	40 ± 13
Range	18, 69
Female sex	45 (60.0)
Duration of diabetes (years)	
Mean ± SD	20.7 ± 10.8
Range	1.8, 54.0
Current insulin pump user	75 (100.0)
Brand of pump most recently used	
Omnipod^a^	74 (98.7)
Other	1 (1.3)
Duration of insulin pump use (years)	
Mean ± SD	8.3 ± 5.9
Range	0.3, 29.2
Number reporting use of CGM at baseline	72 (96.0)

*Note:* Data presented as *n* (%) unless otherwise stated.

Abbreviations: CGM, continuous glucose monitoring; SD, standard deviation.

^a^ Most recently used brand of pump was an Insulet Omnipod (i.e., Omnipod or Omnipod DASH Insulin Management System).

Participants spent a median of 99.7% (Q1‐Q3: 98.9%–99.9%) of the study period using Omnipod 5 in either Manual Mode or Automated Mode. All but 2 participants spent > 90% of the study period in either mode. Participants spent a median of 95.6% (91.5%–98.0%) of the study period with the Omnipod 5 System in Automated Mode. Most of the time (63.2%), participants were using the 110 mg/dL Target Glucose setting. The 120 mg/dL setting was used 23.1% of the time and the 130–150 mg/dL settings were used 13.7% of the time.

The percentage of time spent within target glucose range (70–180 mg/dL) increased by a mean ± SD of 17.9% ± 10.9% (*p* < 0.0001), or 4.3 more hours per day, from baseline (44.6% ± 14.1%) to the extension phase (62.3% ± 9.3%) with a mean glucose change of −27.8 ± 22.3 mg/dL (*p* < 0.0001; Table 2). Concurrently, the percentage of time spent above glucose range (> 180 mg/dL) decreased by 17.7% ± 11.3% (*p* < 0.0001) from baseline (54.1% ± 14.6%) to the extension phase (36.5% ± 9.6%). There were no significant changes in the percentage of time spent in hypoglycemia or total daily insulin use (Table 2).

**TABLE 2:** Extension study outcomes (*N* = 75).

Outcome	Baseline	12‐month extension	Change from baseline	*p*
Time in range 70–180 mg/dL, %	44.6 ± 14.1 44.2 [34.4, 55.6] (*n* = 74)	62.3 ± 9.3 63.7 [56.5, 68.8] (*n* = 75)	17.9 ± 10.9 16.7 [10.7, 25.4] (*n* = 74)	< 0.0001^‡^
Time below range < 54 mg/dL, %	0.26 ± 0.42 0.10 [0.03, 0.32] (*n* = 74)	0.24 ± 0.31 0.16 [0.08, 0.29] (*n* = 75)	−0.02 ± 0.47 0.04 [−0.12, 0.14] (*n* = 74)	0.33^†^
Time below range < 70 mg/dL, %	1.36 ± 1.35 1.01 [0.38, 1.79] (*n* = 74)	1.22 ± 1.01 1.02 [0.55, 1.50] (*n* = 75)	−0.13 ± 1.28 0.04 [−0.63, 0.60] (*n* = 74)	0.80^†^
Time above range > 180 mg/dL, %	54.1 ± 14.6 54.6 [42.5, 64.8] (*n* = 74)	36.5 ± 9.6 35.7 [29.0, 41.7] (*n* = 75)	−17.7 ± 11.3 −15.4 [−25.9, −10.8] (*n* = 74)	< 0.0001^‡^
Mean glucose, mg/dL	198.5 ± 29.0 195.2 [176.0, 220.6] (*n* = 74)	171.0 ± 16.3 169.1 [158.1, 178.9] (*n* = 75)	−27.8 ± 22.3 −23.3 [−43.3, −9.9] (*n* = 74)	< 0.0001^†^
Total daily insulin, units	45.6 ± 16.5 44.0 [33.4, 55.6] (*n* = 72)	45.9 ± 16.1 42.1 [32.1, 57.4] (*n* = 75)	−0.3 ± 7.8 0.4 [−3.3, 3.9] (*n* = 72)	0.72^†^
Total daily insulin, units/kg	0.63 ± 0.19 0.59 [0.50, 0.75] (*n* = 72)	0.61 ± 0.17 0.60 [0.50, 0.72] (*n* = 75)	−0.02 ± 0.10 −0.01 [−0.06, 0.02] (*n* = 72)	0.08^†^

*Note:* Data presented as mean ± SD. Unless otherwise indicated, remaining values are median [Q1, Q3]. To convert the values for glucose from mg/dL to millimoles per litre, multiply by 0.05551. Analyses conducted on a modified intention‐to‐treat dataset.

^†^ *p*‐value determined using two‐sided Wilcoxon signed rank test.

^‡^ *p*‐value determined using unadjusted two‐sided paired *t*‐test.

There were significant improvements in time in range, time above range, and mean glucose when examined by daytime and nighttime (all *p* < 0.0001; Table S3) as well as for participants with baseline HbA_1c_ < 8% (64 mmol/mol), 8%–9% (64–75 mmol/mol), or > 9% (75 mmol/mol) (all *p* < 0.01) (Table S4).

The proportion of participants meeting international targets for both time in range and time below range [15] was 1.4% (95% CI: 0.0, 4.0%) during baseline and 20.0% (95% CI: 10.9, 29.1%) during the extension phase. Nearly all participants met the target of < 4% time below range (94.6% during baseline and 97.3% during the extension; Table S5).

**FIGURE 1:**
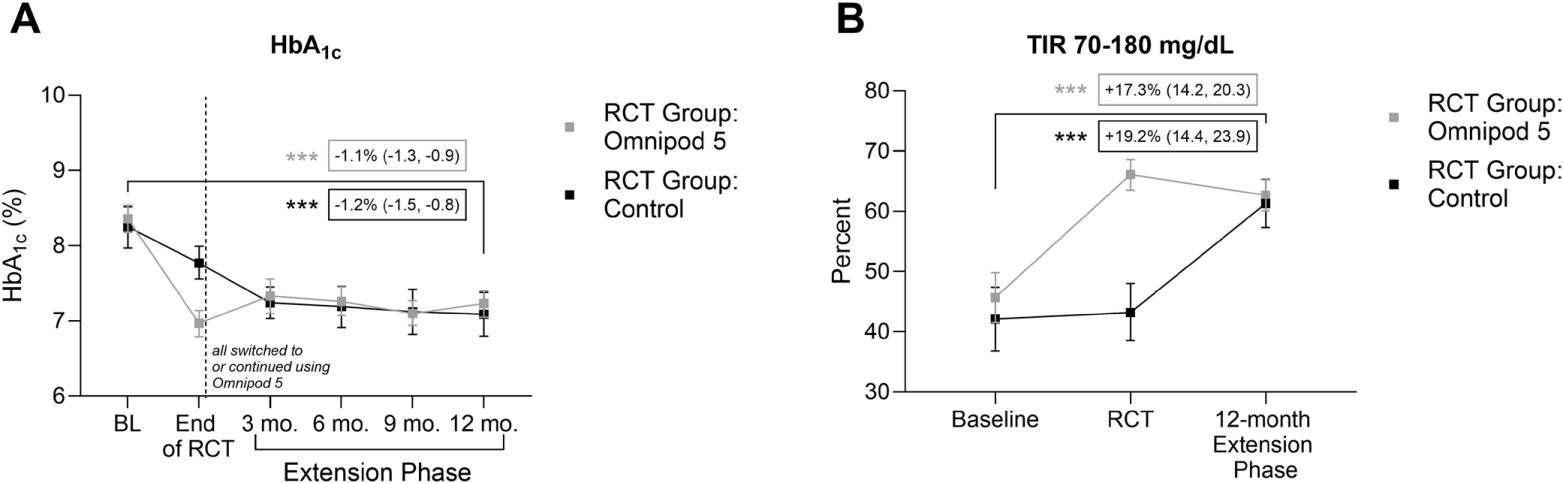
Glycemic improvements with Omnipod 5 use. (A) HbA1c at baseline, end of RCT, and 3, 6, 9, and 12 months during the extension phase, (B) Time in range (TIR) 70–180 mg/dL at baseline, during the RCT, and during the 12‐month extension phase. BL, baseline; RCT, randomized controlled trial. Data presented as mean with 95% confidence interval error bars. ***p < 0.0001.

In the overall cohort, the number of participants with HbA_1c_ < 7% (53 mmol/mol) and < 8% (64 mmol/mol) was 30 and 67 at the end of the extension study, up from 0 and 19 at baseline, respectively (Table 3). The overall change in HbA_1c_ from baseline to end of extension phase was −1.14% (95% CI: −1.31, −0.97; *p* < 0.0001). When the data were stratified by RCT group, consistent HbA_1c_ outcomes were observed among those originally assigned to the Control group of the RCT (−1.16% [−1.52, −0.79] from baseline; *p* < 0.0001; Table 3 and Figure 1A). For those originally assigned to the Intervention group of the RCT, HbA_1c_ improved by −1.41% (−1.61, −1.21) at the end of the RCT (*p* < 0.0001). This was maintained throughout the extension phase with a change of −1.13% (−1.33, −0.94) at the 12‐month endpoint (*p* < 0.0001).

**TABLE 3:** HbA_1c_ at baseline, end of RCT, and at months 3, 6, 9 and 12 of the study extension phase (*n* = 75)*.

Outcome	Baseline	End of RCT	Extension phase			
			3 months	6 months	9 months	12 months
*Overall cohort (N = 75)*						
HbA_1c_, %	8.33 (8.18, 8.48), 8.40 [7.90, 8.80] (*n* = 75)	7.21 (7.04, 7.38), 7.20 [6.70, 7.70] (*n* = 63)	7.30 (7.14, 7.47), 7.20 [6.90, 7.50] (*n* = 51)	7.24 (7.09, 7.39), 7.20 [6.80, 7.60] (*n* = 69)	7.11 (6.97, 7.25), 7.10 [6.65, 7.45] (*n* = 68)	7.18 (7.04, 7.33), 7.20 [6.80, 7.50] (*n* = 74)
HbA_1c_, mmol/mol	68 (66, 69), 68 [63, 73]	55 (53, 57), 55 [50, 61]	56 (55, 58), 55 [52, 58]	56 (54, 57), 55 [51, 60]	54 (53, 56), 54 [49, 58]	55 (53, 57), 55 [51, 58]
Change from baseline, %	—	−1.14 (−1.33, −0.95), −1.20 [−1.70, −0.60] *p* < 0.0001^§^	−1.02 (−1.20, −0.84), −1.00 [−1.40, −0.60] *p* < 0.0001^||^	−1.14 (−1.30, −0.97), −1.20 [−1.50, −0.70] *p* < 0.0001^||^	−1.20 (−1.36, −1.03), −1.20 [−1.70, −0.70] *p* < 0.0001^§^	−1.14 (−1.31, −0.97), −1.20 [−1.70, −0.60] *p* < 0.0001^§^
Change from baseline, mmol/mol	—	−12.5 (−14.5, −10.4), −13.1 [−18.6, −6.6] *p* < 0.0001^§^	−11.1 (−13.1, −9.2), −10.9 [−15.3, −6.6] *p* < 0.0001^||^	−12.5 (−14.2, −10.6), −13.1 [−16.4, −7.7] *p* < 0.0001^||^	−13.1 (−14.9, −11.3), −13.1 [−18.6, −7.7] *p* < 0.0001^§^	−12.5 (−14.3, −10.6), −13.1 [−18.6, −6.6] *p* < 0.0001^§^
HbA_1c_ < 7% (53 mmol/mol), *n* (%)	0 (0.0)	27 (42.9)	17 (33.3)	22 (31.9)	26 (38.2)	30 (40.5)
HbA_1c_ < 8% (64 mmol/mol), *n* (%)	19 (25.3)	52 (82.5)	44 (86.3)	62 (89.9)	62 (91.2)	67 (90.5)
*RCT group: Control (n = 23)*						
HbA_1c_, %	8.25 (7.97, 8.52), 8.20 [7.90, 8.70] (*n* = 23)	7.77 (7.56, 7.99), 7.90 [7.50, 8.10] (*n* = 19)	7.24 (7.03, 7.45), 7.25 [7.05, 7.45] (*n* = 16)	7.19 (6.91, 7.46), 7.10 [7.00, 7.40] (*n* = 21)	7.12 (6.82, 7.42), 7.10 [6.70, 7.40] (*n* = 21)	7.09 (6.80, 7.38), 7.00 [6.80, 7.40] (*n* = 23)
HbA_1c_, mmol/mol	67 (64, 70), 66 [63, 72]	61 (59, 64), 63 [58, 65]	56 (53, 58), 56 [54, 58]	55 (52, 58), 54 [53, 57]	54 (51, 58), 54 [50, 57]	54 (51, 57), 53 [51, 57]
Change from baseline, %	—	−0.52 (−0.82, −0.22), −0.60 [−1.10, −0.10] *p* < 0.0001^§^	−0.99 (−1.24, −0.73), −1.05 [−1.30, −0.60] *p* < 0.0001^§^	−1.06 (−1.36, −0.76), −1.20 [−1.30, −0.70] *p* < 0.0001^§^	−1.13 (−1.53, −0.73), −1.20 [−1.90, −0.60] *p* < 0.0001^§^	−1.16 (−1.52, −0.79), −1.30 [−1.80, −0.60] *p* < 0.0001^§^
Change from baseline, mmol/mol	—	−5.7 (−9.0, −2.4), −6.6 [−12.0, −1.1] *p* < 0.0001^§^	−10.8 (−13.6, −8.0), −11.5 [−14.2, −6.6] *p* < 0.0001^§^	−11.6 (−14.9, −8.3), −13.1 [−14.2, −7.7] *p* < 0.0001^§^	−12.4 (−16.7, −8.0), −13.1 [−20.8, −6.6] *p* < 0.0001^§^	−12.7 (−16.6, −8.6), −14.2 [−19.7, −6.6] *p* < 0.0001^§^
HbA_1c_ < 7% (53 mmol/mol), *n* (%)	0 (0.0)	1 (5.3)	3 (18.8)	5 (23.8)	7 (33.3)	11 (47.8)
HbA_1c_ < 8% (64 mmol/mol), *n* (%)	6 (26.1)	10 (52.6)	15 (93.8)	19 (90.5)	20 (95.2)	22 (95.7)
*RCT group: Intervention (n = 52)*						
HbA_1c_, %	8.36 (8.18, 8.54), 8.40 [7.90, 8.80] (*n* = 52)	6.97 (6.79, 7.14), 6.90 [6.60, 7.50] (*n* = 44)	7.33 (7.10, 7.56), 7.20 [6.90, 7.70] (*n* = 35)	7.26 (7.07, 7.45), 7.30 [6.70, 7.60] (*n* = 48)	7.10 (6.94, 7.27), 7.10 [6.50, 7.50] (*n* = 47)	7.23 (7.05, 7.40), 7.30 [6.70, 7.60] (*n* = 51)
HbA_1c_, mmol/mol	68 (66, 70), 68 [63, 73]	53 (51, 55), 52 [49, 58]	57 (54, 59), 55 [52, 61]	56 (54, 58), 56 [50, 60]	54 (52, 56), 54 [48, 58]	56 (54, 57), 56 [50, 60]
Change from baseline, %	—	−1.41 (−1.61, −1.21), −1.45 [−1.80, −1.00] *p* < 0.0001^§^	−1.03 (−1.27, −0.79), −1.00 [−1.40, −0.60] *p* < 0.0001^||^	−1.17 (−1.37, −0.97), −1.20 [−1.55, −0.90] *p* < 0.0001^||^	−1.23 (−1.40, −1.05), −1.20 [−1.60, −0.90] *p* < 0.0001^§^	−1.13 (−1.33, −0.94), −1.20 [−1.70, −0.60] *p* < 0.0001^§^
Change from baseline, mmol/mol	—	−15.4 (−17.6, −13.2), −15.8 [−19.7, −10.9] *p* < 0.0001^§^	−11.3 (−13.9, −8.6), −10.9 [−15.3, −6.6] *p* < 0.0001^||^	−12.8 (−15.0, −10.6), −13.1 [−16.9, −9.8] *p* < 0.0001^||^	−13.4 (−15.3, −11.5), −13.1 [−17.5, −9.8] *p* < 0.0001^§^	−12.4 (−14.5, −10.3), −13.1 [−18.6, −6.6] *p* < 0.0001^§^
HbA_1c_ < 7% (53 mmol/mol), *n* (%)	0 (0.0)	26 (59.1)	14 (40.0)	17 (35.4)	19 (40.4)	19 (37.3)
HbA_1c_ < 8% (64 mmol/mol), *n* (%)	13 (25.0)	42 (95.5)	29 (82.9)	43 (89.6)	42 (89.4)	45 (88.2)

*Note:* Data presented as mean (95% confidence interval). Unless otherwise indicated, remaining values are median [Q1, Q3].

* Protocol deviations for testing not performed contributed to the missing data.

^§^ Determined using unadjusted two‐sided paired *t*‐test.

^||^ Determined using two‐sided Wilcoxon signed rank test.

Similar results were obtained for time in range 70–180 mg/dL. For the overall cohort, time in range improved by 17.9% (15.3, 20.4) during the extension phase compared to baseline (*p* < 0.0001; Table S6). When the data were stratified by RCT group, time in range did not significantly improve during the RCT for those assigned to the Control group (Table S6 and Figure 1B); however, time in range increased by 19.2% (14.4, 23.9) during the extension phase (*p* < 0.0001). Those assigned to the Intervention group during the RCT maintained their improvements in time in range during the extension phase.

BMI increased by 0.7 kg/m^2^ from baseline to the 6‐month and 12‐month endpoints of the extension study (Table S7). Diabetes Quality of Life‐brief and Hypoglycemia Confidence Scale scores were maintained at 6 months and 12 months of the extension phase compared to RCT baseline (Table 4).

**TABLE 4:** Patient‐reported outcomes at baseline and at months 6 and 12 of the study extension phase.

Outcome	Baseline	6 months	Change from baseline to 6 months	12 months	Change from baseline to 12 months
Hypoglycemia Confidence Scale total score	3.17 ± 0.57 3.22 [2.88, 3.56] (*n* = 75)	3.40 ± 0.51 3.56 [3.11, 3.78] (*n* = 73)	0.25 ± 0.57 0.22 [0.00, 0.56] (*n* = 73)	3.41 ± 0.54 3.56 [3.00, 3.89] (*n* = 72)	0.25 ± 0.56 0.15 [−0.11, 0.61] (*n* = 72)
Diabetes Quality of Life‐brief total score	3.80 ± 0.54 3.80 [3.40, 4.13] (*n* = 75)	4.20 ± 0.43, 4.27 [3.97, 4.47] (*n* = 72)	0.41 ± 0.49 0.40 [0.07, 0.80] (*n* = 72)	4.20 ± 0.45 4.27 [3.93, 4.53] (*n* = 73)	0.40 ± 0.51 0.33 [0.07, 0.73] (*n* = 73)

*Note:* Data presented as mean ± SD. Remaining values are median [Q1, Q3]. Analyses conducted on a modified intention‐to‐treat dataset.

There were nine adverse events (12 per 100 person‐years), which included one episode of prolonged hyperglycemia and eight other non‐glycemic adverse events (Table 5). Seventeen device deficiencies were reported during the extension phase, affecting 13 participants (17.3%). These involved the AID Controller (*n* = 10), the Pod (*n* = 3), the CGM transmitter (*n* = 3), and the CGM sensor (*n* = 1).

**TABLE 5:** Adverse events.

Event	All participants (*n* = 75)
Any adverse event	
No. of events	9
No. of participants with an event (%)	9 (12.0)
No. of events per 100 person‐years	12
Specific events—no. of events/no. of participants (% of participants) [no. events per 100 person‐years]	
Severe hypoglycemia*	0/0 (0.0) [0.0]
Diabetic ketoacidosis^†^	0/0 (0.0) [0.0]
Hypoglycemia^‡^	0/0 (0.0) [0.0]
Hyperglycemia^§^	0/0 (0.0) [0.0]
Prolonged hyperglycemia^||^	1/1 (1.3) [1.3]
Other adverse events^¶^	8/8 (10.7) [10.7]

* Severe hypoglycemia requiring the assistance of another person due to altered consciousness and requiring another person to actively administer carbohydrate, glucagon, or other resuscitative actions.

^†^ Hyperglycemia with the presence of polyuria; polydipsia; nausea or vomiting; serum ketones > 1.5 mmol/L or large/moderate urine ketones; either arterial blood pH < 7.30, venous pH < 7.24, or serum bicarbonate < 15; and treatment provided in a health care facility.

^‡^ Hypoglycemia resulting in an adverse event but otherwise not meeting the definition of severe hypoglycemia.

^§^ Hyperglycemia requiring evaluation, treatment, or guidance from the intervention site or resulting in an adverse event but otherwise not meeting the definition of diabetic ketoacidosis or prolonged hyperglycemia.

^||^ Meter blood glucose measuring ≥ 300 mg/dL for > 1 h and ketones > 1.0 mmol/L.

^¶^ Other nonglycemic adverse events included moderate skin irritation or reaction, moderate atrophic lipodystrophy, as well as other events unrelated to the study procedure or study device.

## 4 Discussion

This study was a 12‐month extension to an RCT investigating the safety and efficacy of the Omnipod 5 System compared to non‐automated insulin pump therapy with CGM among adult participants in the US and France with Type 1 diabetes. In this extension study of participants living in France, the improved efficacy and safety seen with the Omnipod 5 System during the RCT persisted for the length of the 12‐month extension, and was confirmed in the participants of the RCT Control group when moving to the Omnipod 5 System. Specifically, continued Omnipod 5 System use was associated with sustained improvements in time in range (+17.9% or + 4.3 h/day), time above range, and mean glucose, along with few adverse events. HbA_1c_ improvements in the users of the Omnipod 5 System from baseline were sustained from the end of the RCT through to 3, 6, 9, and 12 months of the extension phase.

Mean time in range for the intervention group of the RCT (61.2%; *N* = 132) remained steady at 62.7% (*N* = 52) through the extension phase [11]. Improvements in time in range from baseline were observed regardless of baseline HbA_1c_ level or time of day, and for both those continuing with and those transitioning to the Omnipod 5 System from non‐automated insulin pumps. Those with higher baseline HbA_1c_ levels appeared to experience greater improvements in time in range, akin to those seen in the RCT [11], emphasizing the value of this AID system for participants with a range of HbA_1c_ levels. These improvements, together with the sustained low level of time < 70 mg/dL resulted in a more than 14‐fold increase in the proportion of participants meeting international consensus targets (≥ 70% time in range 70–180 mg/dL and < 4% time below range < 70 mg/dL) and all but two participants had < 4% time below range < 70 mg/dL [15]. Although there were no significant differences in time below range (< 54 mg/dL and < 70 mg/dL) compared to baseline, the glycemic improvements described above were achieved while maintaining a very low percentage of time with glucose in the hypoglycemic range (0.24% ± 0.31% of time < 54 mg/dL and 1.22% ± 1.01% of time < 70 mg/dL). This aligns with the percentage of time below range seen among participants using the Omnipod 5 System in the RCT phase (0.23% ± 0.23% of time < 54 mg/dL and 1.18% ± 0.86% of time < 70 mg/dL) [11].

The sustained improvements in time in range may have implications beyond short‐term glycemic management. Increasing evidence supports an association between greater time in range and lower risk of diabetes‐related microvascular complications, including retinopathy, nephropathy, and neuropathy [16, 17, 18]. Therefore, the durable improvements in time in range observed during the extension phase may be clinically meaningful in the context of longer‐term diabetes outcomes [19], although this study was not designed to assess rates of complication development.

Safety concerns were minimal throughout the extension phase with low rates of prolonged hyperglycemia (1.3 per 100 person‐years) and adverse events overall (12 per 100 person‐years). There were no instances of serious complications like severe hypoglycemia or diabetic ketoacidosis. These rates are lower than diabetic ketoacidosis rates reported in Europe (2.5 per 100 person‐years) [20] and prospectively reported severe hypoglycemia rates in broader real‐world populations (approximately 340–450 events per 100 person‐years across Northern and Eastern Europe) [21]. A comparable study, the Omnipod 5 System pivotal 2‐year extension trial, reported low, but non‐zero rates of severe hypoglycemia and diabetic ketoacidosis (2.04 and 0.24 events per 100 person‐years, respectively). These differences may be reflective of the larger sample size (*n* = 224) and longer study duration [7]. There was also a slight increase in BMI from baseline to 6 and 12 months of the extension phase, but it was unlikely to be clinically meaningful.

The improvements in glycemic measures and low rates of adverse events are echoed in other studies of Omnipod 5 System use among participants with Type 1 diabetes, including long‐term studies of up to 2 years [7, 8], real‐world evidence studies including as many as 69,902 users [9, 10], and single‐arm clinical trials [5, 6]. Most similarly, extension studies from a single‐arm pivotal trial of the Omnipod 5 System in children, adolescents, and adults similarly demonstrated maintained improvements in HbA_1c_ and time in range for up to 2 years [7, 8]. Evidence from extension studies, systematic reviews, and meta‐analyses that include other AID systems also aligns with these findings, showing short and long‐term glycemic benefits [22, 23, 24, 25], further emphasizing the value of using such systems as a first‐line therapy for those with Type 1 diabetes [2, 4]. While the findings of this extension study do not represent a novel efficacy signal for AID use, they do provide important confirmatory evidence that the benefits observed during an RCT were sustained during extended use in a routine clinical setting.

Patient‐reported outcomes for the Omnipod 5 System in this study were not tested statistically but were similar to the observed adjusted differences from the RCT phase, which were clinically meaningful improvements in the intervention compared to the control group (Diabetes Quality of Life‐brief: 0.43 [95% CI: 0.31, 0.55], HCS: +0.20 [95% CI: 0.06, 0.34], *p* < 0.05). All but one of the 76 eligible participants opted into the extension phase, and approximately two‐thirds had already been using the System as part of the RCT. Moreover, the System was operated in Automated Mode for most of the time (median 95.6%), and only one participant did not complete the extension phase. Together, these findings show support for user satisfaction and long‐term adoption of the Omnipod 5 System. Future analyses of Omnipod 5 System use in broader populations and in real‐world settings may further characterize long‐term utilization patterns, glycemic outcomes, user‐selected settings (e.g., Target Glucose), and patient‐reported outcomes over extended periods of use.

Notable strengths of this work include the long duration of the study in which participants used the Omnipod 5 System for 12 months, providing insights into the long‐term benefits of the System's use. This long duration may be more reflective of real‐world conditions and can account for short‐term improvements that may result from the novelty of initiating a new system. Furthermore, the higher levels of baseline HbA_1c_ are more reflective and generalizable to the real‐life burden of Type 1 diabetes [26]. However, interpretation of the findings should consider that participants were adults in France with Type 1 diabetes who were already using insulin pump therapy and had baseline HbA_1c_ values between 7.0% and 11.0%. Consequently, these results may not be fully generalizable to individuals using multiple daily injections, those with substantially lower or higher HbA_1c_ values, or other populations not represented in the study. Notably, a slight decrease in time in range among Omnipod 5 system users from the end of the RCT to end of the extension phase may reflect less intensive monitoring during the extension phase and underscores the importance of ongoing healthcare professional support to maintain optimal glycemic outcomes with AID therapy.

In conclusion, the initial short‐term glycemic, safety, and patient‐reported outcomes observed for the Omnipod 5 System in a 13‐week RCT were maintained over the course of a 12‐month extension phase among adults with Type 1 diabetes in France. Importantly, there were no reports of severe hypoglycemia, diabetic ketoacidosis, or safety concerns associated with the use of the System over the extended period. This work supplements a growing body of literature demonstrating the sustained benefits of AID system use in this population.

## Author Contributions

**Jean‐Pierre Riveline:** methodology, investigation, writing – original draft, writing – review and editing. **Eric Renard:** methodology, investigation, writing – original draft, writing – review and editing. **Trang T. Ly:** conceptualization, methodology, writing – original draft, writing – review and editing. **Alfred Penfornis:** methodology, investigation, writing – original draft, writing – review and editing. **Charles Thivolet:** methodology, investigation, writing – original draft, writing – review and editing. **Ruth S. Weinstock:** methodology, investigation, writing – original draft, writing – review and editing.

## Funding

Support for this study and the article processing charge was provided by Insulet Corporation.

## Conflicts of Interest

E.R. declares consultant/speaker fees from A. Menarini Diagnostics, Abbott, Air Liquide SI, AstraZeneca, Becton, Dickinson and Company, Boehringer Ingelheim, Cellnovo, Dexcom, Eli Lilly, Hillo, Insulet Corporation, Johnson & Johnson (Animas, LifeScan), Medtronic, Medirio, Novo Nordisk, Roche, and Sanofi Aventis and research support from Abbott, Dexcom Inc., Insulet Corporation, Roche, and Tandem Diabetes Care. R.S.W. participated in clinical trials, through her institution, sponsored by Amgen, Diasome, Insulet Corporation, Eli Lilly, MannKind, and Tandem and has used Dexcom devices obtained at a reduced cost for clinical studies. A.P. reports personal fees from Abbott Diabetes Care, Dexcom, Diabeloop, Insulet Corporation, Eli Lilly, Novo Nordisk, Medtronic, and Sanofi and is an advisory board member for Insulet Corporation and Medtronic. J.‐P.R. is an advisory panel member for Sanofi Aventis MSD, Eli Lilly, Novo Nordisk, AstraZeneca, Abbott, Dexcom, Alphadiab, Air Liquide, and Medtronic and has received research funding from Abbott, Air Liquide, Sanofi, Eli Lilly, Insulet Corporation, and Novo Nordisk. C.T. reports personal fees from Abbott Diabetes Care, Glooko, Eli Lilly, Novo Nordisk, Medtronic, and Sanofi and is an advisory board member for Insulet Corporation and Medtronic. T.T.L. is a full‐time employee of and owns stock in Insulet Corporation. No other potential conflicts of interest relevant to this article were reported.

## Supporting information


**Figure S1:** CONSORT diagram of the randomized controlled trial (RCT) and subsequent 12‐month extension phase.

**Table S1:** Eligibility and exclusion criteria.
**Table S2:** Schedule of participant visits.
**Table S3:** Glycemic outcomes by time of day (*n* = 75).
**Table S4:** Glycemic outcomes by baseline HbA1c (*n* = 75).
**Table S5:** Proportion of participants meeting time in range (70–180 mg/dL) and time below range (< 70 mg/dL) targets* (*n* = 75).
**Table S6:** Time in range 70–180 mg/dL at baseline, during RCT, and during the 12‐month extension phase.
**Table S7:** Body mass index at baseline and Months 6 and 12 of the study extension phase (*n* = 75).

## Data Availability

The datasets generated during and/or analysed in the current study are not publicly available.
