# Supplementary material for: Extended Use of the Omnipod 5 Automated Insulin Delivery System in Adults With Type 1 Diabetes: 12‐Month Extension of a Randomized Controlled Trial
Source: Endocrinol Diabetes Metab. 2026 Aug 31;9(5):e70321. doi: 10.1002/edm2.70321 (PMC13528233; doi:10.1002/edm2.70321)
Supplement: Supplementary file 1 [file EDM2-9-e70321-s002.docx]

**Supplementary Material**

This appendix has been provided by the authors to give readers additional information about their work.

Supplement to: Extended Use of the Omnipod^®^ 5 Automated Insulin Delivery System in Adults with Type 1 Diabetes: 12-Month Extension of a Randomized Controlled Trial

Contents

[OP5-003 Research Group	2](#_Toc232438481)

[Table S1. Eligibility and exclusion criteria	3](#_Toc232438482)

[Table S2. Schedule of participant visits	5](#_Toc232438483)

[Table S3. Glycemic outcomes by time of day (n=75)	6](#_Toc232438484)

[Table S4. Glycemic outcomes by baseline HbA_1c_ (n=75)	7](#_Toc232438485)

[Table S5. Proportion of participants meeting time in range (70-180 mg/dL) and time below range (<70 mg/dL) targets^*^ (n=75)	8](#_Toc232438486)

[Table S6. Time in range 70-180 mg/dL at baseline, during RCT, and during the 12-month extension phase	9](#_Toc232438487)

[Table S7. Body mass index at baseline and months 6 and 12 of the study extension phase (n=75)	10](#_Toc232438488)

[Supplementary Figure 1. CONSORT diagram of the randomized controlled trial (RCT) and subsequent 12-month extension phase	11](#_Toc232438489)

# OP5-003 Research Group

Clinical site principal investigators (P.I.) are noted.

**Montpellier University Hospital, Montpellier, France**

Eric Renard MD PhD (PI), Anne Farret MD (Co-I), Orianne Villard MD (Co-I), Manal Al Masri

**SUNY Upstate Medical University, Syracuse, New York, USA**

Ruth S. Weinstock MD PhD (PI), Suzan Bzdick RN CDCES CCRC

**Centre Hospitalier Sud Francilien, Université Paris-Saclay, Corbeil-Essonnes, France**

Alfred Penfornis MD PhD (PI), Catherine Petit (Co-I), Marcelle Siadoua

**Hôpital Lariboisière, APHP, Université Paris-Cité, Paris, France**

Jean-Pierre Riveline MD PhD (PI), Jean-François Gautier MD PhD (Co-I), Tiphaine Vidal-Trecan MD (Co-I), Jean-Baptiste Julla MD (Co-I), Charline Potier, Djamila Bellili

**DIAB-eCARE Diabetes Center, Lyon, France**

Charles Thivolet MD PhD (PI), Sylvie Villar Fimbel MD, Redhouane Hami

**Fortrea Inc.**

Kaisa Kivilaid MS

**Insulet Corporation, Acton, Massachusetts, USA**

Trang T. Ly MBBS FRACP PhD, Bonnie Dumais RN, Todd Vienneau BSc, Lauren M. Huyett PhD, Lindsey R. Conroy PhD

# Table S1. Eligibility and exclusion criteria

***Inclusion Criteria***

Participants must meet all of the following criteria to be included in the study:

1. Age at time of consent 18-70 years of age;
2. Diagnosed with type 1 diabetes for at least 1 year. Diagnosis is based on investigator’s clinical judgment;
3. On pump therapy for ≥3 months prior to screening and familiar with pump therapy concepts such as basal and bolus insulin delivery, and carbohydrate counting. Participants using automated insulin delivery devices, including devices with predictive low glucose suspend, in the 3 months prior to screening, will be excluded from participating;
4. Glycated hemoglobin 7.0-11.0% (53 to 97 mmol/mol) by point-of-care taken at screening visit;
5. Willing to use and obtain U-100 insulin (either insulin aspart (Novolog, NovoRapid), or insulin lispro (Humalog, Admelog)), as the primary insulin treatment;
6. Must have a smartphone that supports download of the Dexcom app and participants must be willing to use the app throughout the study;
7. Investigator has confidence that the participant can safely operate all study devices and can adhere to the protocol;
8. Willing to wear the system continuously throughout the study;
9. Willing and able to sign the Informed Consent Form; and
10. Able to read and understand English.^*^

***Exclusion Criteria***

Participants who meet any of the following criteria will be excluded from the study:

1. Any medical condition, such as untreated malignancy, unstable cardiac disease, unstable or end-stage renal failure, eating disorders, or other conditions which in the opinion of the investigator, would put the participant at an unacceptable safety risk;
2. History of severe hypoglycemia in the past 6 months;
3. History of diabetic ketoacidosis in the past 6 months, unrelated to an intercurrent illness or infusion set failure;
4. Blood disorder or dyscrasia within 3 months prior to screening, including use of hydroxyurea, which in the investigator’s opinion could interfere with determination of glycated hemoglobin;
5. Currently on systemic steroids or intends to receive systemic steroid treatment in the next 6 months, including stable treatment for adrenal insufficiency. Inhaled, ophthalmic, topical, joint injection, and other locally applied steroids are allowed;
6. Unable to tolerate adhesive tape or has any unresolved skin condition in the area of sensor or pump placement;
7. Use of non-insulin anti-hyperglycemic medication other than metformin, in the 12 weeks prior to the Baseline Visit. Participants taking metformin should remain on a steady dose during study participation;
8. Pregnant or lactating, or is a woman of childbearing potential and not on an acceptable form of birth control (acceptable forms of contraception include abstinence, barrier methods such as condoms, hormonal contraceptives, intrauterine device, surgical sterilization such as tubal ligation or hysterectomy, or vasectomized partner);
9. Participation in another clinical study using an investigational drug or device within 30 days or 5 half-lives (whichever is longer) prior to screening, or intends to participate in any other study during this study period;
10. Unable to follow clinical protocol for the duration of the study or is otherwise deemed unacceptable to participate in the study per the investigator’s clinical judgment;
11. Participant is an employee of Insulet, an Investigator or Investigator’s study team, or immediate family member of any of the aforementioned;
12. Participants who have had a pancreas or pancreatic islet transplantation;^*^
13. Impaired hypoglycemia awareness (Clarke score ≥ 4);^*^
14. Participant suffering from a serious illness or undergoing treatment that may impact the physiology of diabetes (i.e. resulting in interactions with glucose and/or insulin likely to interfere with the medical device);^*^
15. Presence of non-stabilized retinopathy or painful neuropathy;^*^
16. Hearing and/or vision impairment that would interfere with recognition of all functions of the Omnipod 5 System, including alerts, alarms, and reminders;^*^
17. Added procedures to manage hypoglycemia and procedures for specific blood glucose checks using the study-issued blood glucose meter; and^*^
18. Participants under court protection.^*^

^*^ Criteria added for the 12-month extension phase. All other eligibility criteria were derived from the 13-week randomized controlled trial.

# Table S2. Schedule of participant visits

|  | **12-Month (Extension Phase)** | | | | | | | | | |
| --- | --- | --- | --- | --- | --- | --- | --- | --- | --- | --- |
| **Visit Number**^*^ | **9** | **10^†^** | **11^†^** | **12^†^** | **13** | **14** | **15** | **16** | **UV** | **EW^\|\|^** |
| Study day | ~0 | 1 | 14 | 45 | 90 | 180 | 270 | 360 |  |  |
| Remote (R) or office (O) visit | O | R/O^§^ | R/O^§^ | R/O^§^ | O | O | O | O | R/O^§^ | O |
| **Laboratory Assessments** |  |  |  |  |  |  |  |  |  |  |
| HbA_1c_ (Central Lab) | X |  |  |  | X | X | X | X |  | X |
| Pregnancy test (urine dipstick) | X |  |  |  |  |  |  |  |  |  |
| **Clinical Assessments** |  |  |  |  |  |  |  |  |  |  |
| Review of concomitant medications | X | X | X | X | X | X | X | X |  | X |
| Weight |  |  |  |  |  | X |  | X |  | X |
| Vital signs^‡^ | X | X | X | X | X | X | X | X |  | X |
| Review of Medical History | X |  |  |  |  |  |  |  |  |  |
| Assessment of adverse events | X | X | X | X | X | X | X | X | X | X |
| **Study Devices** |  |  |  |  |  |  |  |  |  |  |
| Omnipod 5 System device training^¶^ | X |  |  |  |  |  |  |  |  |  |
| Stop CGM data collection |  |  |  |  |  |  |  | X |  | X |
| Return Omnipod 5 System |  |  |  |  |  |  |  | X |  | X |
| Return BG meter, ketone meter, and study CGM |  |  |  |  |  |  |  | X |  | X |
| Completion of the DQOL-brief questionnaire and HCS questionnaire |  |  |  |  |  | X |  | X |  | X |
| Complaints/device deficiencies since last visit | X | X | X | X | X | X | X | X | X | X |
| BG/ketone meter data review by clinician |  | X | X | X | X | X | X | X |  | X |
| Pump and CGM data review by clinician |  | X | X | X | X | X | X | X | X | X |

Abbreviations: BG, blood glucose; CGM, continuous glucose monitor; DQOL-brief, diabetes quality of life brief; EW, early withdrawal; HCS, hypoglycemia confidence scale; UV, unscheduled visits.

^*^ Visits up to 8 were part of the standard therapy and randomized controlled trial (RCT) phase and have been previously published (1).

^†^ Visits 10, 11, and 12 were for participants who did not use the Omnipod 5 System during the RCT (control group).

^‡^ Vital signs include heart rate, blood pressure, temperature, and breathing rate and should be performed at all in person visits.

^§^ Visits identified as “R/O” can either be conducted in person at the clinical site or remotely. Visits identified as “O” can only be conducted in person at the clinical site. Vital signs were not measured at any visit conducted remotely.

^||^ Early withdrawal visit will only occur prior to visit 16.

^¶^ If participants were new to the Omnipod 5 System.

# Table S3. Glycemic outcomes by time of day (n=75)

| **Outcome** | **Baseline (n=74)** | **12-month extension (n=75)** | **Change from Baseline (n=74)** | **P-value** |
| --- | --- | --- | --- | --- |
| **Daytime** | | | | |
| Time in range 70-180 mg/dL, % | 46.5 ± 14.9,  46.3 [36.5, 58.9] | 61.6 ± 10.1,  63.4 [54.5, 68.7] | 15.4 ± 11.0,  16.5 [6.6, 23.4] | <0.0001^‡^ |
| Time below range <54 mg/dL, % | 0.24 ± 0.42,  0.09 [0.00, 0.29] | 0.23 ± 0.29,  0.14 [0.07, 0.27] | -0.01 ± 0.45,  0.05 [-0.15, 0.17] | 0.21^†^ |
| Time below range <70 mg/dL, % | 1.39 ± 1.37,  0.94 [0.51, 1.92] | 1.26 ± 1.04,  1.05 [0.51, 1.68] | -0.13 ± 1.22,  -0.09 [-0.74, 0.63] | 0.68^†^ |
| Time above range >180 mg/dL, % | 52.2 ± 15.6,  53.0 [38.7, 62.6] | 37.1 ± 10.5,  35.6 [29.9, 44.7] | -15.2 ± 11.5,  -16.0 [-23.8, -6.1] | <0.0001^‡^ |
| Mean glucose, mg/dL | 194.9 ± 29.3,  194.1 [168.8, 208.1] | 171.6 ± 17.6,  169.6 [159.7, 183.7] | -23.6 ± 21.8,  -24.4 [-38.4, -3.9] | <0.0001^‡^ |
| **Nighttime** | | | | |
| Time in range 70-180 mg/dL, % | 39.0 ± 17.0,  39.2 [25.8, 51.5] | 64.2 ± 12.1,  65.8 [54.8, 71.9] | 25.2 ± 16.0,  21.4 [13.5, 35.2] | <0.0001^‡^ |
| Time below range <54 mg/dL, % | 0.32 ± 0.69,  0.00 [0.00, 0.31] | 0.27 ± 0.40,  0.17 [0.07, 0.29] | -0.05 ± 0.77,  0.07 [-0.10, 0.17] | 0.16^†^ |
| Time below range <70 mg/dL, % | 1.26 ± 1.84,  0.53 [0.00, 1.75] | 1.11 ± 1.11,  0.84 [0.47, 1.24] | -0.16 ± 2.01,  0.26 [-0.43, 0.64] | 0.30^†^ |
| Time above range >180 mg/dL, % | 59.7 ± 17.5,  59.4 [44.7, 73.7] | 34.7 ± 12.3,  33.4 [26.4, 41.8] | -25.1 ± 16.5,  -21.0 [-35.8, -13.6] | <0.0001^†^ |
| Mean glucose, mg/dL | 209.3 ± 36.9,  202.3 [179.0, 227.9] | 169.3 ± 18.6,  167.5 [155.9, 179.2] | -40.0 ± 32.7,  -29.6 [-62.2, -18.2] | <0.0001^†^ |

Data presented as mean ± SD. Unless otherwise indicated, remaining values are median [Q1, Q3]. Analyses conducted on a modified intention-to-treat dataset.

^†^ P value determined using two-sided Wilcoxon signed rank test.

^‡^ P-value determined using unadjusted two-sided paired t-test.

# Table S4. Glycemic outcomes by baseline HbA_1c_ (n=75)

| **Outcome** | **Baseline** | **12-month extension** | **Change from Baseline** | **P-value** |
| --- | --- | --- | --- | --- |
| **Baseline HbA_1c_ <8% (<64 mmol/mol), n** | 18 | 19 | 18 |  |
| Time in range 70-180 mg/dL, % | 53.3 ± 9.7,  55.1 [42.7, 59.0] | 67.6 ± 6.8,  67.6 [64.3, 72.3] | 15.2 ± 10.1,  15.2 [5.7, 24.1] | <0.0001^‡^ |
| Time below range <54 mg/dL, % | 0.46 ± 0.68,  0.15 [0.05, 0.71] | 0.25 ± 0.34,  0.13 [0.09, 0.28] | -0.21 ± 0.65,  0.01 [-0.35, 0.11] | 0.55^†^ |
| Time below range <70 mg/dL, % | 1.90 ± 1.96,  0.96 [0.75, 2.32] | 1.34 ± 1.18,  0.96 [0.76, 1.46] | -0.54 ± 1.53,  -0.25 [-1.04, 0.60] | 0.35^†^ |
| Time above range >180 mg/dL, % | 44.8 ± 10.2,  44.0 [37.7, 54.3] | 31.0 ± 7.0,  31.4 [26.1, 34.3] | -14.7 ± 10.3,  -13.5 [-24.7, -6.2] | <0.0001^‡^ |
| Mean glucose, mg/dL | 180.0 ± 16.4,  180.6 [168.2, 192.6] | 161.8 ± 11.6,  163.8 [153.7, 168.5] | -19.7 ± 15.7,  -20.3 [-31.4, -8.5] | <0.0001^‡^ |
| **Baseline HbA_1c_ 8-9% (64-75 mmol/mol), n** | 47 | 47 | 47 |  |
| Time in range 70-180 mg/dL, % | 43.5 ± 14.3,  44.2 [34.2, 54.8] | 61.3 ± 9.8,  62.2 [55.1, 68.8] | 17.8 ± 11.1,  16.1 [10.7, 26.7] | <0.0001^‡^ |
| Time below range <54 mg/dL, % | 0.21 ± 0.29,  0.10 [0.00, 0.33] | 0.26 ± 0.32,  0.19 [0.09, 0.30] | 0.05 ± 0.42,  0.07 [-0.10, 0.17] | 0.07^†^ |
| Time below range <70 mg/dL, % | 1.30 ± 1.11,  1.15 [0.38, 1.96] | 1.30 ± 0.98,  1.26 [0.55, 1.60] | 0.00 ± 1.27,  0.07 [-0.69, 0.72] | 0.77^†^ |
| Time above range >180 mg/dL, % | 55.2 ± 14.8,  55.3 [42.7, 64.9] | 37.4 ± 10.1,  36.5 [30.3, 43.7] | -17.8 ± 11.6,  -15.1 [-27.0, -10.8] | <0.0001^‡^ |
| Mean glucose, mg/dL | 200.6 ± 29.1,  203.6 [176.0, 221.1] | 172.1 ± 17.2,  169.6 [161.7, 181.5] | -28.4 ± 22.5,  -24.3 [-47.3, -7.1] | <0.0001^‡^ |
| **Baseline HbA_1c_ >9% (>75 mmol/mol), n** | 9 | 9 | 9 |  |
| Time in range 70-180 mg/dL, % | 32.7 ± 9.2,  28.8 [27.7, 39.5] | 56.2 ± 5.8,  58.4 [52.9, 60.5] | 23.6 ± 10.0,  23.3 [16.0, 32.2] | 0.004^†^ |
| Time below range <54 mg/dL, % | 0.13 ± 0.10,  0.10 [0.08, 0.13] | 0.11 ± 0.10,  0.08 [0.03, 0.10] | -0.02 ± 0.14,  -0.03 [-0.10, 0.03] | 0.65^†^ |
| Time below range <70 mg/dL, % | 0.59 ± 0.43,  0.38 [0.33, 0.96] | 0.56 ± 0.44,  0.52 [0.27, 0.59] | -0.03 ± 0.45,  -0.07 [-0.40, 0.16] | 0.91^†^ |
| Time above range >180 mg/dL, % | 66.7 ± 9.3,  70.1 [59.5, 72.3] | 43.2 ± 6.1,  40.5 [39.0, 47.0] | -23.5 ± 10.3,  -23.5 [-31.7, -15.6] | 0.004^†^ |
| Mean glucose, mg/dL | 224.9 ± 25.4,  225.2 [213.2, 238.0] | 184.2 ± 8.6,  181.2 [178.1, 186.7] | -40.6 ± 27.4,  -36.1 [-59.1, -22.3] | 0.004^†^ |

Data presented as mean ± SD. Unless otherwise indicated, remaining values are median [Q1, Q3]. Analyses conducted on a modified intention-to-treat dataset. N values reflect available CGM data per column.

^†^ P value determined using two-sided Wilcoxon signed rank test.

^‡^ P-value determined using unadjusted two-sided paired t-test.

# Table S5. Proportion of participants meeting time in range (70-180 mg/dL) and time below range (<70 mg/dL) targets^*^ (n=75)

| **Outcome** | **Baseline**^†^ | **12-month extension phase** |
| --- | --- | --- |
| **≥70% Time in range 70-180 mg/dL** | 1.4% [0.0%, 4.0%]  (n=1) | 20.0% [10.9%, 29.1%]  (n=15) |
| **<4% Time below range <70 mg/dL** | 94.6% [89.4%, 99.7%]  (n=70) | 97.3% [93.7%, 100%]  (n=73) |
| **≥70% Time in range 70-180 mg/dL and <4% time below range <70 mg/dL** | 1.4% [0.0%, 4.0%]  (n=1) | 20.0% [10.9%, 29.1%]  (n=15) |

^*^ Recommended CGM targets (≥70% time in range [70–180 mg/dL] and <4% time below range [<70 mg/dL]) are based on the International Consensus on Time in Range (2).

^†^ Data presented as % of participants [95% CI]; n=number of participants.

# Table S6. Time in range 70-180 mg/dL at baseline, during RCT, and during the 12-month extension phase

| **Outcome** | **Baseline** | **During RCT** | **12-month**  **extension phase** |
| --- | --- | --- | --- |
| **Overall cohort (N=75)** | | | |
| Time in Range 70-180 mg/dL, % | 44.6 (41.3, 47.8),  44.2 [34.4, 55.6]  (n=74) | 59.1 (55.8, 62.4),  62.7 [49.2, 69.8]  (n=75) | 62.3 (60.1, 64.4),  63.7 [56.5, 68.8]  (n=75) |
| Change from baseline, % | -- | 14.6 (11.5, 17.7),  14.5 [5.2, 25.6]  P<0.0001^§^ | 17.9 (15.3, 20.4),  16.7 [10.7, 25.4]  P<0.0001^§^ |
| **RCT Group: Control (n=23)** | | | |
| Time in Range 70-180 mg/dL, % | 42.1 (36.8, 47.4),  41.4 [31.4, 50.8]  (n=23) | 43.2 (38.5, 48.0),  43.3 [33.6, 50.9]  (n=23) | 61.3 (57.3, 65.3),  61.6 [52.9, 68.0]  (n=23) |
| Change from baseline, % | -- | 1.1 (-2.8, 5.0),  2.2 [-8.9, 5.7]  P=0.57^§^ | 19.2 (14.4, 23.9),  18.4 [12.1, 30.8]  P<0.0001^§^ |
| **RCT Group: Intervention (n=52)** | | | |
| Time in Range 70-180 mg/dL, % | 45.7 (41.5, 49.8),  45.0 [34.8, 57.1]  (n=51) | 66.1 (63.5, 68.6),  66.2 [60.2, 72.6]  (n=52) | 62.7 (60.1, 65.4),  64.1 [57.3, 69.3]  (n=52) |
| Change from baseline, % | -- | 20.7 (17.9, 23.5),  22.7 [12.6, 27.4]  P<0.0001^§^ | 17.3 (14.2, 20.3),  16.1 [9.5, 24.6]  P<0.0001^§^ |

Data presented as mean (95% confidence interval). Unless otherwise indicated, remaining values are median [Q1, Q3].

^§^ determined using unadjusted two-sided paired t-test

# Table S7. Body mass index at baseline and months 6 and 12 of the study extension phase (n=75)

| **Outcome** | **Baseline**^†^ | **6 months** | **12 months** |
| --- | --- | --- | --- |
| **Body mass index,**  **kg/m^2^** | 25.0 ± 3.8, 24.4 [22.1, 27.0]  (n=75) | 25.7 ± 3.9, 25.2 [22.3, 27.7]  (n=66) | 25.8 ± 4.2,  25.0 [22.5, 28.4]  (n=74) |
| **Change in body**  **mass index, kg/m^2^** | -- | 0.7 ± 1.6, 0.5 [0.0, 1.3]^\|\|^ | 0.7 ± 1.9, 0.8 [-0.2, 1.8]^\|\|^ |

Data presented as mean ± SD. Unless otherwise indicated, remaining values are median [Q1, Q3].

^†^ Baseline values were collected before the randomized controlled trial.

^||^ P<0.0001 determined using two-sided Wilcoxon signed rank test.

# Supplementary Figure 1. CONSORT diagram of the randomized controlled trial (RCT) and subsequent 12-month extension phase

**References**

1.	Renard E, Weinstock RS, Aleppo G, Bode BW, Brown SA, Castorino K, et al. Efficacy and safety of a tubeless AID system compared with pump therapy with CGM in the treatment of type 1 diabetes in adults with suboptimal glycemia: A randomized, parallel-group clinical trial. Diabetes Care. 2024;47(12):2248–57.

2.	Battelino T, Danne T, Bergenstal RM, Amiel SA, Beck R, Biester T, et al. Clinical targets for continuous glucose monitoring data interpretation: recommendations from the international consensus on time in range. Diabetes Care. 2019;42(8):1593–603.
